# Anna vs. Judith: A randomized comparison of AI-delivered psychodynamic and cognitive behavioral therapies for social anxiety disorder^☆^

**DOI:** 10.1016/j.invent.2026.100960

**Published:** 2026-06-13

**Authors:** Jón Ingi Hlynsson, Jakob Mechler, Karin Lindqvist, Gerhard Andersson, Per Carlbring

**Affiliations:** a Department of Psychology, Stockholm University, Stockholm, Sweden; b Department of Psychology, Uppsala University, Uppsala, Sweden; c The Erica Foundation, Sweden; d Department of Behavioural Sciences and Learning, Linköping University, Linköping, Sweden; e Department of Clinical Neuroscience, Karolinska Institute, Stockholm, Sweden; f Lusófona University, Digital Human-Environment Interaction Labs, Lisboa, Portugal; g School of Psychology, Korea University, Seoul, 02481, South Korea; h Department of Biomedical and Clinical Sciences, Linköping University, Linköping, Sweden

**Keywords:** Social anxiety disorder, Artificial intelligence, Cognitive behavioral therapy, Psychodynamic therapy, Neurodivergence

## Abstract

Artificial intelligence (AI) offers a potential solution to the scalability limits of internet-based psychological interventions. This randomized controlled trial evaluated two smartphone-based, AI-delivered interventions for social anxiety disorder (SAD): Psychodynamic Therapy (AI-PDT) and Cognitive Behavioral Therapy (AI-CBT). One hundred and two adults with SAD were randomized (1:1:1) to AI-PDT, AI-CBT, or a waitlist control for a 4-week daily intervention. The primary outcome was social anxiety severity measured by the Social Phobia Inventory, and analyzed using Linear Mixed Models. Both interventions yielded significant, moderate reductions in symptoms from baseline to post-treatment, with within group effect sizes of *d* = 0.79 (AI-PDT) and *d* = 0.72 (AI-CBT), and with no significant differences observed between the two active treatments. However, the between-group difference against the waitlist control at post-treatment was not significant (AI-PDT: *p* = .204, *d* = 0.43; AI-CBT: *p* = .727, *d* = 0.18), possibly due to substantial improvement in the waitlist condition (*d* = 0.54). At 1-month follow-up, the difference widened: AI-PDT became significantly superior to waitlist (*d* = 0.65), while AI-CBT did not (*d* = 0.51). Therapeutic alliance was established early in both conditions and remained stable throughout treatment, suggesting that AI-delivered interventions can foster a therapeutic alliance regardless of theoretical orientation. Secondary regression analysis revealed that a co-occurring diagnosis of ADHD or autism spectrum disorder significantly predicted poorer outcomes. These findings suggest that the comparative efficacy of AI-guided interventions may increase over time and are capable of fostering moderate symptom reductions, though future iterations require adaptation to better support neurodivergent users.

## Highlights

• AI-guided PDT and CBT yielded large within-group effect sizes for social anxiety.
• High waitlist response reduced the power to detect between-group differences.
• Co-occurring ADHD and autism diagnoses predicted poorer treatment outcomes.
• Therapeutic alliance with the AI was established early and remained stable.

## 1 Introduction

Social anxiety disorder (SAD) is a debilitating mental health condition that typically emerges during childhood or adolescence (Solmi et al., 2022), with a lifetime prevalence estimate of 10.7% and an estimated 12-month prevalence of 7.4% (Kessler et al., 2012). Individuals suffering from social anxiety believe they will behave ineptly and unacceptably in social situations, leading to catastrophic consequences like loss of status, worth, and rejection (Clark and Wells, 1995). Thus, social situations frequently trigger fear and anxiety that is disproportionate to any actual threat. Therefore, individuals with SAD either avoid these situations entirely or endure them with severe distress (American Psychiatric Association, 2022).

Given the high prevalence of SAD in the general population, and the nature of the problem being associated with active avoidance of social situations, researchers and clinicians have developed ways to administer treatment remotely (see e.g., Andersson et al., 2006; Furmark et al., 2009; Ma et al., 2020; Mechler et al., 2024a, Mechler et al., 2024b). Results indicate that both guided and unguided self-help for social anxiety is effective in reducing social anxiety symptoms (Guo et al., 2021), albeit larger symptom reductions are generally found when the self-help material is guided by a clinician (see e.g., Furmark et al., 2009; Mechler et al., 2024a, Mechler et al., 2024b).

Internet-delivered psychological interventions have seen a significant increase in adoption and innovation since the late 1990s, particularly accelerated by the COVID-19 pandemic (De Witte et al., 2021). Designed to parallel conventional face-to-face therapy in length and content (Seiferth et al., 2023), these interventions range from live, real-time video therapy sessions, such as telepsychiatry via platforms like Zoom, to self-guided digital self-help programs, with or without asynchronous human guidance (Andersson and Carlbring, 2022). Internet-delivered therapy has gained increasing empirical support. For instance, guided Internet-delivered Cognitive Behavioral Therapy (ICBT) has been found to be as effective as traditional face-to-face therapy for a wide range of psychiatric and somatic disorders in a recent meta-analysis (Hedman-Lagerlöf et al., 2023). Internet-delivered Psychodynamic Therapy (IPDT) has been less researched, but has shown efficacy for social anxiety in adults (Hedman et al., 2011; Johansson et al., 2017; Mechler et al., 2024a) as well as for depression in adolescents and adults (Johansson et al., 2013; Mechler et al., 2022).

While ICBT has been shown to yield outcomes comparable to traditional face-to-face therapy (Hedman-Lagerlöf et al., 2023), meta-analytic findings indicate that between 35% and 50% of individuals suffering from SAD do not achieve a clinical response to treatment with current state-of-the-art treatments such as CBT and pharmacotherapy (Leichsenring and Leweke, 2017). Despite ICBT's greater scalability over conventional methods (Andersson et al., 2019), its scalability is still limited by the necessity for therapists to devote approximately 15 min per week to provide individualized feedback to each patient (Nordgren et al., 2014).

The integration of Artificial Intelligence (AI) into internet interventions invites the possibility to increase their scalability even further, without increasing clinical burden on therapists whom otherwise would have devoted time to provide individualized feedback (Löchner et al., 2025). Advances in AI technology, particularly large language models (LLMs) and generative AI, offer the potential to automate aspects of therapeutic feedback, potentially mirroring—or even enhancing—the therapeutic guidance provided by human therapists. This could take the form of virtual psychoeducational coaches available around the clock or AI entities capable of delivering sophisticated, clinically informed feedback, such as Socratic questioning to aid clients in analyzing negative thought patterns or disputing maladaptive core beliefs (Carlbring et al., 2023; Wiederhold, 2025). As such, integrating AI into internet interventions can provide patients with immediate, on-demand support outside standard business hours, effectively bypassing traditional waitlists.

While AI presents promising advancements, it simultaneously poses challenges and ethical concerns, particularly its lack of emotional intelligence, crucial in therapeutic settings where genuine empathy and personal experience are important (Svensson et al., 2025). Although capable of mimicking empathy and handling complex language, AI's inability to fully grasp human emotions may result in misunderstandings of non-verbal cues and subtleties in communication. Specifically, while AI can successfully simulate cognitive empathy by generating validating responses, it fundamentally lacks affective empathy (i.e., the capacity to genuinely share in a patient's emotional experience; Rubin et al., 2024). This ‘empathy gap’ means that AI may fail to recognize the unspoken emotional weight behind a patient's words (Salil et al., 2025), potentially leading to responses that feel superficial or inappropriately upbeat, thereby risking a rupture in the digital therapeutic alliance.

Despite potential for agreement on treatment tasks and goals, the development of a bond with AI remains limited (Miloff et al., 2020). That said, some evidence suggests that users still form a ‘digital working alliance’ with AI agents (Darcy et al., 2021). Interestingly, the transparently artificial nature of this bond can sometimes serve as an advantage; knowing the AI cannot judge them, patients may feel a reduced fear of evaluation, fostering high levels of self-disclosure even in the absence of a traditional human connection (Darcy et al., 2021). Client preferences also significantly influence the acceptance of AI in therapy. This builds on the long-observed trend of individuals preferring to self-manage symptoms rather than seeking traditional therapy (Andrade et al., 2014), with many now turning to digital guidance to achieve that autonomy. Today, this preference positions AI less as a traditional therapy replacement and more as a vital, low-threshold early intervention within flexible healthcare systems (Löchner et al., 2025). However, while the theoretical foundations for AI-guided interventions are being established, empirical research directly comparing different AI-delivered psychological intervention remains scarce. To our knowledge, no trial has yet conducted a head-to-head comparison of different AI-delivered therapy interventions.

The aim of the present randomized control trial was to evaluate the short-term efficacy of two smartphone-based, AI-guided psychotherapies for social anxiety by comparing an intervention aligned with cognitive behavioral therapy (AI-CBT), an intervention aligned with psychodynamic therapy (AI-PDT), and a waitlist control over 4 weeks. We hypothesized that AI-PDT and AI-CBT would both lead to greater reductions in social anxiety severity than waitlist at post-assessment and follow-up. Furthermore, we hypothesized comparable efficacy between the AI-PDT and AI-CBT conditions, mirroring the general equivalence often observed between these modalities in internet interventions. Secondary analyses were exploratory and included an evaluation of which baseline factors predict treatment outcome, whether the treatment had an effect on secondary outcomes such as depressive and general anxiety symptom severity, whether participants differ in satisfaction and perceived benefit across arms, and critically, the extent to which a therapeutic working alliance can be established and maintained with an AI assistant. Finally, we investigated whether response and remission rates differ between the active interventions and waitlist.

## 2 Method

### 2.1 Participants and recruitment

Participants were recruited online through a secure website that outlined the study's aims and organization (Vlaescu et al., 2026). Recruitment took place in April 2025, in Sweden, and was primarily conducted via social media but also spread through word of mouth. The study was approved by the Swedish Ethical Review Authority (Dnr: 2025-01603-02; 2025-03910-02). The trial was registered on ClinicalTrials.gov (NCT07533812). Participants were randomized to one of the intervention groups or to the waitlist control group according to a 1:1:1 randomization ratio. To ensure allocation concealment, this randomization sequence was embedded directly within the secure website's backend. Participants were automatically allocated by the computerized system only after their eligibility was confirmed, ensuring the assignment sequence remained completely concealed from all investigators. Individuals excluded from the study due to an elevated risk of suicide were contacted by a licensed psychologist that explained why they had been excluded and provided them with information about where they could seek appropriate help. Ongoing evaluation of suicide risk was not conducted during the active treatment phase, and participant safety during the intervention thus relied on the rigorous baseline exclusion of at-risk individuals. Primary data were collected at three time points: at screening (pre-treatment), after treatment completion (post-treatment), and at a 1-month follow-up. In addition, weekly symptom tracking was initiated one day post-randomization (prior to meaningful treatment engagement) and continued every seven days across the four-week intervention.

#### 2.1.1 Eligibility criteria

Participants were eligible for inclusion in the study if they met the following criteria: Adults (≥ 18 years) presenting with a primary diagnosis of SAD as indicated by scoring ≥30 on the Liebowitz Social Anxiety Scale Self-report (LSAS-SR; Liebowitz, 1987), having access to a computer/smartphone/tablet with internet connection, and being able to read, write, and speak Swedish. Participants were excluded if they fulfilled any of the following exclusion criteria: scores ≤29 on LSAS-SR, substantial risk of suicide (i.e., clear intent and/or plans) and/or suicide attempts in the last three months as assessed by the Columbia-Suicide Severity Rating Scale Self-Report (C-SSRS; Posner et al., 2011), primary diagnosis of severe major depression (scores of ≥20 on Patient Health Questionnaire [PHQ-9]; Kroenke et al., 2001), ongoing participation in other psychological treatment(s), and psychotropic medication not stable during the last month.

### 2.2 Treatment interventions

In both AI-PDT and AI-CBT, the AI assistant functioned as a digital coach that offers reflective questions, reminders, and encouragement. Participants had access to their allocated AI assistant on an ‘on-demand’ basis, albeit with an upper limit of 75 messages per day. This is similar in many ways to how modern applications for physical training or meditation function; they offer a structured progression with personal adaptation without requiring human resources for each participant (e.g., Chowdhury and Chowdhury, 2025). Technically, the AI-assistant utilizes a multi-agent orchestration architecture paired with Retrieval-Augmented Generation (RAG) to ensure high clinical fidelity. This system employs specialized agents—including a long-term planner to maintain treatment adherence and a supervisory “critic” agent—that validate every response against evidence-based protocols (see below) and safety guardrails before output. Participants were not told whether they had been assigned to AI-PDT or AI-CBT and the AI assistants were named Samuel and Simon, respectively. As such, participants were blind to their allocated treatment condition. Crucially, all conversational inputs and outputs were encrypted locally on participants' devices to ensure maximal privacy and anonymity. While this privacy-by-design approach secured the confidentiality of the therapeutic space, it inherently precluded the central logging of messages. Consequently, we cannot analyze exact usage metrics, such as the frequency of contact or specific conversational content. To maintain clinical oversight despite this decentralized encryption, participants were provided an option to flag any generated response as inappropriate. Flagging a response immediately triggered a manual review by a licensed psychologist affiliated with the research project. Notably, no inappropriate responses were reported during the intervention period. Claude 3.7 Sonnet was used for all agents.

#### 2.2.1 AI-Guided Psychodynamic Therapy (AI-PDT)

AI-PDT treatment was based on Malan's triangle of conflict, where unconscious affects are thought to trigger anxiety, leading to defenses such as projection, which are thought to exacerbate social anxiety symptoms. Treatment aims to increase capacity for self-observation, facilitate experience of underlying anxiety-laden affects, and help participants recognize repetitive maladaptive relational patterns (see e.g., Mechler et al., 2024b). Instead of human therapist support, participants receive support from an AI assistant programmed to encourage reflection on key concepts from the treatment material, in a similar way to how a well-informed therapist would do.

#### 2.2.2 AI-Guided Cognitive Behavioral Therapy (AI-CBT)

AI-CBT was based on established CBT principles for social anxiety (Andersson et al., 2006; Furmark et al., 2019), optimized for smartphone format. It includes psychoeducation about anxiety, cognitive restructuring, exposure, and behavioral experiments. Here too, human therapist support is replaced with an AI assistant that encourages reflection and exercise completion.

#### 2.2.3 Waitlist-control group

Participants in the control group received no active treatment during the first 4 weeks but underwent the same assessments as the treatment groups. After the study period, participants were offered a choice between CBT or PDT treatment but not followed up specifically.

### 2.3 Measures

#### 2.3.1 Screening and eligibility measures

##### 2.3.1.1 Liebowitz Social Anxiety Scale – Self-Report (LSAS-SR)

The Liebowitz Social Anxiety Scale (LSAS-SR) is a 24-item self-report questionnaire, intended to quantify social anxiety symptom severity (Baker et al., 2002; Fresco et al., 2001; Liebowitz, 1987). Notably, the LSAS-SR has been psychometrically validated for digital administration, demonstrating equivalent reliability and construct validity to traditional paper-and-pencil formats (Hedman et al., 2010). Items represent common daily scenarios (e.g., “going to a party”) that respondents rate for both the level of fear it elicits and the degree to which it is avoided. Although the factor structure of the LSAS-SR has been debated (Baroni et al., 2023), the measure is commonly conceptualized as comprising two domains: Fear and Avoidance. Each item is scored on a 0–3 scale for both subscales, yielding a total possible score of 0–144. In this study, internal consistency for the total scale was excellent, Cronbach's α = 0.95 [95% CI: 0.94, 0.96]. For the Fear and Avoidance subscales, reliability was α = 0.91 [95% CI: 0.89, 0.93]. Model-based reliability was also high, McDonald's ω = 0.96, suggesting a strong general factor underlying the scale. LSAS-SR scores were measured at pre-treatment to confirm eligibility and establish baseline clinical severity, ensuring sample comparability with prior clinical trials in this domain.

##### 2.3.1.2 Columbia-Suicide Severity Rating Scale (C-SSRS)

The Columbia-Suicide Severity Rating Scale (C-SSRS) assesses suicidal ideation and behavior and was administered during pre-treatment screening to evaluate eligibility in the present study (Posner et al., 2011).

#### 2.3.2 Primary outcome measure

##### 2.3.2.1 Social Phobia Inventory (SPIN)

The Social Phobia Inventory (SPIN) is a 17-item self-report questionnaire designed to assess the severity of social anxiety disorder symptoms (Connor et al., 2000). The SPIN was specifically selected as the primary outcome given its high sensitivity to clinical change over time. Items capture three interrelated domains: fear of social situations (e.g., fear of being criticized, speaking to strangers, or being the center of attention), avoidance of such situations, and physiological discomfort (e.g., blushing, sweating, trembling). Each item is rated on a 0–4 scale (0 = *not at all* to 4 = *extremely*), yielding a total score range of 0–68, with higher scores indicating greater symptom severity. Despite debates about the underlying factor structure voiced by previous research, evidence does support the use of a total score for the SPIN (for an overview see e.g., Campbell-Sills et al., 2015). In this study, internal consistency for the total scale was excellent, Cronbach's α = 0.90 [95% CI: 0.88, 0.92]. Model-based reliability was also high, McDonald's ω = 0.92, suggesting a strong general factor underlying the measure. SPIN scores were measured at pre-treatment baseline, during post-assessment and follow-up assessments, as well as weekly. While formal inferential modelling of weekly SPIN measurements are outside the scope of the present paper, weekly scores are presented descriptively to visually illustrate the general course of symptom change throughout the intervention.

#### 2.3.3 Secondary outcome measures

##### 2.3.3.1 Patient Health Questionnaire-9 (PHQ-9)

The Patient Health Questionnaire-9 (PHQ-9) is a self-report measure composed of nine items assessing the severity of depressive symptoms (Kroenke et al., 2001). Designed to capture all nine symptoms of major depression (Hlynsson et al., 2025), the PHQ-9 routinely demonstrates good accuracy and discrimination ability in both clinical settings and the general population (Hlynsson and Carlbring, 2024; Martin-Key et al., 2022). Each item is rated on a 0–3 scale reflecting symptom frequency over the past two weeks, with total scores ranging from 0 to 27; a total score of 10 or higher is considered a diagnostic indicator for depression (Kroenke et al., 2001, Kroenke et al., 2010). In this study, the internal consistency reliability for the PHQ-9 was good, Cronbach's α = 0.87 [95% CI: 0.84, 0.90]. Model-based reliability was also high, McDonald's ω = 0.90, suggesting a strong general factor underlying the scale. PHQ-9 scores were measured during pre-treatment screening.

##### 2.3.3.2 Generalized Anxiety Disorder 7-item scale (GAD-7)

The GAD-7 is a self-report questionnaire consisting of seven items that quantify anxiety symptom severity (Spitzer et al., 2006). Although initially designed to index symptoms of generalized anxiety disorders, the GAD-7 is sensitive to various anxiety disorders (Kroenke et al., 2010) and has demonstrated good accuracy and discrimination ability in both clinical settings and the general population (Hlynsson and Carlbring, 2024; Martin-Key et al., 2022). Each item is rated on a 0–3 scale reflecting symptom frequency over the past two weeks, with total scores ranging from 0 to 21; a total score of 8 or higher is considered a diagnostic indicator for an anxiety disorder (Luo et al., 2019). In this study, the internal consistency reliability for the GAD-7 was good, Cronbach's α = 0.90 [95% CI: 0.87, 0.92]. Model-based reliability was also high, McDonald's ω = 0.93, suggesting a strong general factor underlying the measure. GAD-7 scores were measured at pre-treatment baseline, during post-assessment and follow-up assessments.

##### 2.3.3.3 The Negative Effects Questionnaire (NEQ)

The Negative Effects Questionnaire (NEQ) is a 20-item self-report measure that evaluates the negative effects of psychological treatment (Rozental et al., 2016). The NEQ total score indicates the average negative impact of treatment (Rozental et al., 2016, Rozental et al., 2019). It should be noted that there is no current consensus on interpreting scores from self-report measures of negative effects in psychological treatments (Rozental and Řiháček, 2025). Nevertheless, presenting mean frequencies and standard deviations can facilitate comparisons across different samples. Negative effects were assessed at post-assessment.

##### 2.3.3.4 Session Alliance Inventory (SAI)

The Session Alliance Inventory (SAI) is a 6-item measure of working alliance (Falkenström et al., 2015). The SAI assesses the therapeutic bond and agreement on tasks/goals for the current session on a 6-point Likert scale ranging from 0 (“not at all”) to 5 (“completely”). The SAI is typically treated as unidimensional and has previously demonstrated excellent internal consistency (α ≈ 0.90) and strong measurement invariance across sessions in psychotherapy samples (Falkenström et al., 2015). In this study, internal consistency reliability for the SAI was good at all instances of measurement (Cronbach's α between 0.89 and 0.96). Session alliance was measured weekly during the treatment phase.

##### 2.3.3.5 Client Satisfaction Questionnaire (CSQ)

The Client Satisfaction Questionnaire (CSQ) is an 8-item self-report measure that assesses client satisfaction with treatment (Attkisson and Greenfield, 2004). Each item on the CSQ is rated on a scale from 1 to 4, with total scores ranging from 8 to 32; higher scores are indicative of greater satisfaction with treatment. Previous studies have demonstrated that the CSQ is a reliable and valid measure of client satisfaction across different languages and administration methods (Attkisson and Greenfield, 2004; Kelly et al., 2018; Vázquez et al., 2019). In this study, the internal consistency reliability for the CSQ was good, Cronbach's α = 0.94 [95% CI: 0.92, 0.95]. Model-based reliability was also high, McDonald's ω = 0.96, suggesting a strong general factor underlying the measure. Treatment satisfaction was measured at post-assessment.

##### 2.3.3.6 Qualitative User Experience

To capture ongoing perceptions of the AI assistant and identify elements participants found useful or challenging, open-ended qualitative feedback was collected weekly. Participants were asked to provide free-text responses to four prompts: (1) “Which Aspect of the AI Coaching Was Most Useful for You This Week?”; (2) “What Was Your Biggest Frustration or Challenge Using the App This Week?”; (3) “What Feature or Change Would Make You Use the App More Regularly?”; and (4) “Is There Anything Else You Would Like to Add?”. Qualitative user experience is presented as a supplement to the main quantitative outcome analyses and is descriptive and exploratory in its scope.

### 2.4 Data analysis

Data analysis was conducted using R (R Core Team, 2021) and the *lme4* package (Bates et al., 2015). Descriptive statistics for demographic and clinical characteristics were summarized using means and standard deviations (SD) for continuous variables, and frequencies and percentages for categorical variables.

To evaluate the efficacy of the interventions in accordance with the Intention-to-Treat (ITT) principle, differences in the primary outcome (SPIN) were analyzed using Linear Mixed Models (LMM). This approach allowed for the inclusion of all randomized participants regardless of data loss at subsequent time points, under the assumption that data were Missing at Random (MAR). As the MAR assumption is fundamentally untestable, the use of LMM represents the standard and most robust approach for mitigating bias associated with longitudinal dropout. The model specified a random intercept for each participant to account for repeated measures dependency, with fixed effects for Group (AI-PDT, AI-CBT, Waitlist), Time (Baseline, Post-treatment, Follow-up), and the Group × Time interaction. Because this was an early-stage trial of novel AI-delivered interventions, we powered the study to detect the moderate-to-large effect sizes typical of unguided digital interventions (cf. Furmark et al., 2009; Mechler et al., 2024a). Specifically, 34 participants per arm provides 80% power (alpha = 0.05) to detect moderate-to-large between-group differences. To ensure model convergence given the sample size (n ≈ 34 per arm at each timepoint), we adopted a parsimonious random-intercept-only structure (Matuschek et al., 2017). Time was modeled as a discrete factor to capture non-linear change trajectories at each assessment point, rather than assuming a linear slope. Final parameters were estimated using Restricted Maximum Likelihood (REML) to provide unbiased variance estimates.

The primary hypothesis tests compared the estimated marginal means of the active treatment groups (AI-PDT and AI-CBT) against the Waitlist control at the post-treatment assessment. To control for the family-wise error rate when comparing two treatment arms against a single control, *p*-values were adjusted using Dunnett's method via the *emmeans* package (Lenth et al., 2025). Between-group comparisons against the Waitlist were restricted to the post-treatment time point, and long-term maintenance of gains was evaluated using within-group contrasts (Baseline vs. Follow-up) for the active conditions, and was visualized using raincloud plots (Allen et al., 2021).

Effect sizes were calculated to quantify the magnitude of change. Both within-group and between-group effect sizes (Cohen's *d*) were derived by dividing the estimated mean change or mean difference from baseline to post-treatment or follow-up by the pooled baseline standard deviation at baseline (Morris, 2008). Effect sizes were interpreted as small (*d* = 0.20), medium (*d* = 0.50), and large (*d* = 0.80; Cohen, 1977). Between-group estimates were also reported as the mean difference in SPIN scores derived from the LMM, accompanied by 95% confidence intervals (CIs).

Secondary analyses assessed the effect of treatment on depressive and anxiety symptom severity (PHQ-9 and GAD-7) using the same methodology as for the primary outcome. Furthermore, clinical significance was evaluated by assessing the response and remission rates. Response was defined as a reduction of ≥30% in SPIN total score from baseline, and remission was defined as a post-treatment SPIN score below 19 (cf. Connor et al., 2000). Differences in response and remission rates between groups were analyzed using chi-square tests, and differences in treatment satisfaction and negative effects were analyzed using independent samples *t*-tests.

To identify predictors of treatment outcome, multiple linear regression analyses were conducted for the active treatment groups. To preserve statistical power given the sample size, the model was restricted to four predictors chosen based on clinical and theoretical relevance to digital psychotherapy outcomes: (1) baseline social anxiety (SPIN) to control for initial severity; (2) age, to account for potential generational differences in digital literacy and technology use; (3) early therapeutic alliance (SAI rating at week 4), a well-established common factor predicting treatment success; and (4) the presence of a neurodevelopmental diagnosis (ADHD/ASD), given emerging evidence that neurodivergent populations may respond differently to standard digital protocols. Participant treatment preference match was not included as a predictor, as participants were blinded to their treatment allocation, neutralizing the expectancy effects typically associated with receiving a preferred therapy. A sensitivity power analysis was conducted to ensure the model was adequately powered to detect meaningful effect sizes given the sample size constraints.

Finally, negative effects of treatment and treatment satisfaction were explored at post-treatment. All statistical tests were two-tailed with an alpha level of 0.05, unless otherwise adjusted for multiple comparisons.

## 3 Results

### 3.1 Descriptive statistics

**Fig. 1:**
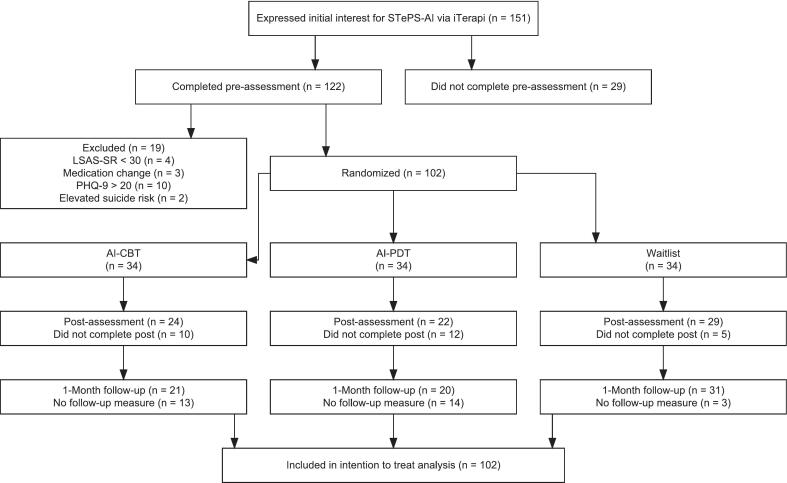
CONSORT diagram.

The CONSORT diagram for the study is presented in Fig. 1. In April 2025, 122 potential study participants completed initial screening questionnaires; 102 (84%) were eligible and subsequently randomized to one of the intervention groups or waitlist condition. Table 1 presents descriptive statistics and summarizes demographical data for the sample. The sample reported high levels of social anxiety at baseline, with a mean LSAS-SR score of 73.04 (SD = 21.67).

**Table 1:** Baseline demographic characteristics.

Characteristic	Overall (*N* = 102)	AI-CBT (*n* = 34)	AI-PDT (*n* = 34)	Waitlist (*n* = 34)
Age				
Mean in years (SD)	41.0 (11.3)	41.6 (10.9)	39.5 (10.8)	41.8 (12.2)
Range	18–74	22–74	21–60	18–63
Gender, n (%)				
Female	74 (73%)	26 (76%)	25 (74%)	23 (68%)
Male	27 (26%)	8 (24%)	9 (26%)	10 (29%)
Other/unsure	1 (1.0%)	0 (0%)	0 (0%)	1 (2.9%)
Living situation, n (%)				
Alone	32 (31%)	8 (24%)	14 (41%)	10 (29%)
With partner/friend(s)	55 (54%)	20 (59%)	17 (50%)	18 (53%)
Other	15 (15%)	6 (18%)	3 (8.8%)	6 (18%)
Education level, n (%)				
Primary school	4 (3.9%)	0 (0%)	1 (2.9%)	3 (8.8%)
High school	23 (23%)	10 (29%)	7 (21%)	6 (18%)
Vocational/folk school	19 (19%)	5 (15%)	9 (26%)	5 (15%)
University	56 (55%)	19 (56%)	17 (50%)	20 (59%)
Occupation, n (%)				
Working	60 (59%)	21 (62%)	21 (62%)	18 (53%)
Studying	18 (18%)	3 (8.8%)	5 (15%)	10 (29%)
Parental leave	1 (1.0%)	1 (2.9%)	0 (0%)	0 (0%)
Sick leave	7 (6.9%)	2 (5.9%)	1 (2.9%)	4 (12%)
Unemployed	12 (12%)	6 (18%)	5 (15%)	1 (2.9%)
Other	4 (3.9%)	1 (2.9%)	2 (5.9%)	1 (2.9%)
Comorbidities, n (%)				
ADHD/ASD Diagnosis⁎	31 (30%)	10 (29%)	11 (32%)	10 (29%)
Treatment preference, n (%)				
Strong preference (PDT)	18 (18%)	8 (24%)	5 (15%)	5 (15%)
Moderate preference (PDT)	16 (16%)	7 (21%)	6 (18%)	3 (8.8%)
Neutral	47 (46%)	13 (38%)	19 (56%)	15 (44%)
Moderate preference (CBT)	5 (4.9%)	0 (0%)	2 (5.9%)	3 (8.8%)
Strong preference (CBT)	14 (14%)	4 (12%)	2 (5.9%)	8 (24%)
Unsure/other	2 (2.0%)	2 (5.9%)	0 (0%)	0 (0%)

*Note.* Data are presented as mean (standard deviation) or *n* (%). ADHD = Attention-Deficit/Hyperactivity Disorder; ASD = Autism Spectrum Disorder; CBT = Cognitive Behavioral Therapy; PDT = Psychodynamic Therapy. Percentages may not total 100 due to rounding.

⁎ Information on ADHD/ASD diagnoses were obtained through a binary self-reported question.

Observed values for the clinical measures at all three time points are presented in Table 2.

**Table 2:** Descriptive statistics for clinical measures by group and time.

Measure	Group	Pre-treatment	Post-assessment	Follow-up
SPIN-17 (Social Anxiety)	Waitlist	41.44 (11.34), *n* = 34	35.72 (15.10), *n* = 29	36.68 (15.34), *n* = 31
	PDT	39.18 (11.61), *n* = 34	31.05 (12.83), *n* = 22	28.05 (11.48), *n* = 20
	CBT	41.41 (11.60), *n* = 34	31.88 (12.98), *n* = 24	27.61 (13.41), *n* = 23
PHQ-9 (Depression)	Waitlist	7.50 (4.60), *n* = 34	9.10 (6.14), *n* = 29	8.94 (6.48), *n* = 31
	PDT	9.68 (5.01), *n* = 34	7.95 (5.37), *n* = 22	5.35 (3.62), *n* = 20
	CBT	9.00 (5.16), *n* = 34	6.83 (5.37), *n* = 23	5.10 (4.23), *n* = 20
GAD-7 (Anxiety)	Waitlist	9.12 (5.04), *n* = 34	8.00 (5.04), *n* = 29	8.00 (5.60), *n* = 31
	PDT	10.82 (5.95), *n* = 34	7.00 (4.73), *n* = 22	5.85 (4.43), *n* = 20
	CBT	9.56 (5.51), *n* = 34	7.09 (6.12), *n* = 23	5.00 (4.03), *n* = 20

### 3.2 Primary outcome analysis

We began by investigating potential predictors of study attrition. Attrition rates differed significantly across treatment conditions (χ2(2) = 9.60, *p* = .008), with higher retention observed in the waitlist group compared to the active treatment arms. However, missingness at follow-up was not significantly predicted by baseline SPIN symptom severity (*p* = .521). The presence of group-dependent missingness was inherently accounted for in the primary LMM analyses.

To confirm the integrity of the randomization process, demographic characteristics and pre-treatment scores on clinical measures were compared across the three groups. One-way ANOVAs (for continuous variables) and Chi-square/Fisher's exact tests (for categorical variables) revealed no significant differences between the groups regarding age, gender, living situation, education level, neurodevelopmental diagnoses, SPIN, PHQ-9, or GAD-7 scores at pre-treatment baseline (all *p* > .05). However, a small but statistically significant pre-treatment difference was observed for the LSAS scores (*F*(2, 99) = 3.17, *p* = .046). Upon further examination, a correlation analysis of our pre-treatment data revealed a strong and significant relationship between the baseline SPIN and LSAS measures (*r* = 0.76, *p* < .001). As such, we elected not to include pre-treatment LSAS as an additional covariate in our primary outcome analysis for post-treatment and follow-up SPIN scores, since the primary model already controls for pre-treatment SPIN scores (which shares approximately 58% of its variance with the LSAS). We thus concluded that the randomization had yielded comparable groups and proceeded with the primary linear mixed modelling.

Linear Mixed Model analyses (ITT) indicated that at post-treatment, participants in the AI-PDT condition scored an average of 5.28 points lower on the SPIN than the Waitlist control (see Table 3). However, this difference was not statistically significant after adjusting for multiple comparisons (*p* = .204; Cohen's *d* = 0.43). Similarly, the difference between AI-CBT and Waitlist at post-treatment was not significant (*p* = .727; Cohen's *d* = 0.18).

**Table 3:** Between-group differences in social anxiety (SPIN) at Post-treatment and Follow-up (Mixed Model Analysis).

Timepoint	Contrast	Estimate (SE)	95% CI	*t*	*p*-Value†
**Post-treatment**	AI-PDT vs Waitlist	−5.28 (3.32)	[−12.74, 2.18]	−1.59	0.204
	AI-CBT vs Waitlist	−2.16 (3.29)	[−9.54, 5.21]	−0.66	0.727
**Follow-up**	AI-PDT vs Waitlist	−7.87 (3.34)	[−15.38, −0.38]	−2.36	0.038⁎
	AI-CBT vs Waitlist	−6.22 (3.28)	[−13.59, 1.15]	−1.90	0.111

*Note.* Estimates derived from Linear Mixed Models (MMRM) accounting for missing data (Intention-to-Treat). Negative values indicate lower anxiety in the treatment group relative to Waitlist. CI = Confidence Interval.

† *P*-values adjusted for multiple comparisons using Dunnett's method.

⁎ *p* < .05.

At the 1-month follow-up, the mean difference between AI-PDT and Waitlist increased to −7.87 points, reaching statistical significance (*p* = .038; Cohen's *d* = 0.65). While the difference between AI-CBT and Waitlist also widened to −6.22 points at follow-up, it remained non-significant (*p* = .111; Cohen's *d* = 0.51).

Irrespective of group differences, participants in the active treatment conditions demonstrated substantial reductions in social anxiety symptoms over time. The AI-PDT group showed a significant reduction from baseline to post-treatment (Estimate = −9.64, *p* < .001), corresponding to a moderate within-group effect size (Cohen's *d* = 0.79). Similarly, the AI-CBT group reported significant improvements from baseline to post-treatment (Estimate = −8.76, *p* < .001), yielding a moderate effect size (*d* = 0.72). Notably, the Waitlist group also demonstrated a significant, albeit smaller, reduction in symptoms during the waiting period (Estimate = −6.63, *p* < .001), corresponding to a medium effect size (*d* = 0.54).

**Fig. 2:**
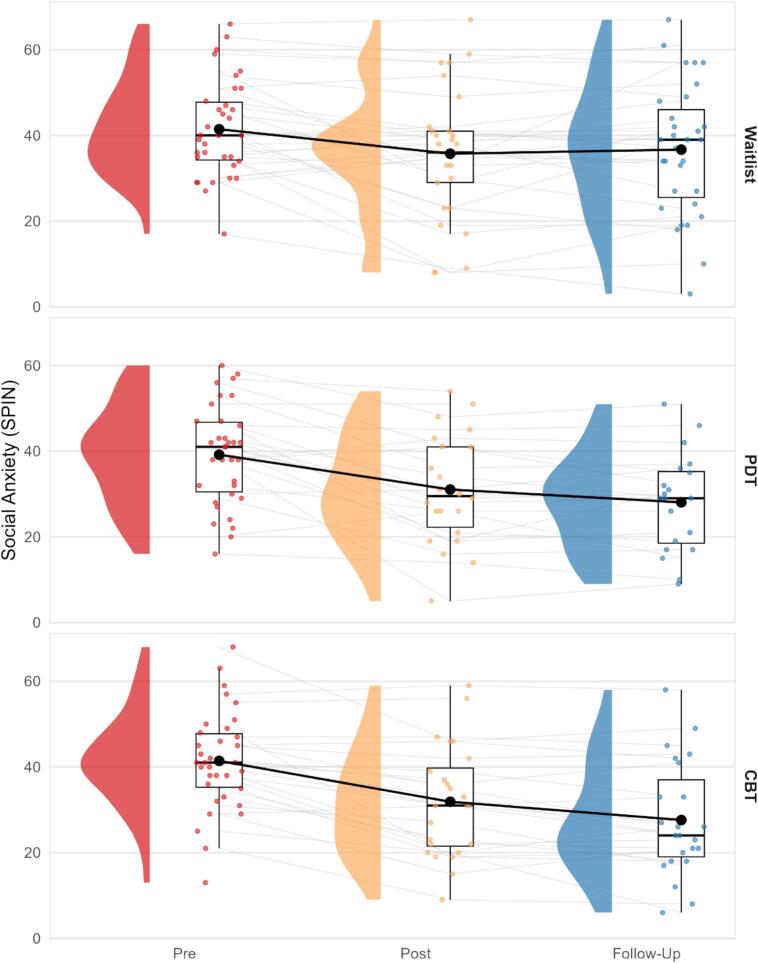
Raincloud plots of Social Anxiety (SPIN) trajectories across treatment conditions. Half-violin plots denote probability density, and central box plots show median and interquartile ranges. Individual scores are represented by jittered points with gray lines connecting within-person changes from pre-treatment through 1-month follow-up.

Finally, regarding the comparative effectiveness of the active interventions, there were no statistically significant differences between AI-PDT and AI-CBT at post-treatment (Estimate = −3.12, *t* = −0.92, *p* = .63) nor at the 1-month follow-up (Estimate = −1.65, *t* = −0.48, *p* = .88). This indicates that both AI-guided modalities produced similar symptom trajectories over the study period. Fig. 2 displays the trajectory of social anxiety symptoms by group. Estimated marginal means and standard errors are presented in Table S1 in the *Supplementary Material*.

### 3.3 Secondary outcome analysis

Analyses of secondary outcomes followed the same Linear Mixed Model strategy as the primary outcome. For depressive symptoms (PHQ-9), AI-CBT demonstrated a significant within-group reduction from baseline to post-treatment (*d* = 0.32; *p* = .039), while the reduction in AI-PDT did not reach statistical significance (*d* = 0.18; *p* = .350). There were no statistically significant differences between either active treatment and the Waitlist control at the post-treatment assessment (AI-PDT vs. Waitlist: Estimate = −0.09, *d* = 0.01, *p* = .995; AI-CBT vs. Waitlist: Estimate = −1.66, *d* = 0.27, *p* = .390).

For generalized anxiety (GAD-7), both active groups improved significantly over time. AI-PDT showed a moderate within-group effect (*d* = 0.71; p < .001), and AI-CBT showed a small effect (*d* = 0.43; *p* = .010). However, between-group comparisons against the Waitlist at post-treatment were not significant (AI-PDT vs. Waitlist: Estimate = −0.65, *d* = 0.12, *p* = .846; AI-CBT vs. Waitlist: Estimate = −0.34, *d* = 0.06, *p* = .948). Descriptive statistics for these measures are provided in Table 2.

### 3.4 Response and remission rates

Response (defined as >30% reduction in SPIN score) and remission (SPIN <19) rates were calculated at post-treatment and 1-month follow-up. At post-treatment assessment, there were no statistically significant differences in response rates between groups (*p* = .90), with response achieved by 40.9% [95% CI: 21.5%, 63.3%] in the AI-PDT group (*n* = 9), 37.5% [95% CI: 19.6%, 59.2%] in the AI-CBT group (*n* = 9), and 34.5% [95% CI: 18.6%, 54.3%] in the Waitlist group (*n* = 10). Remission rates were similarly low and non-significant at post-treatment assessment (*p* = .80), ranging from 8.3% [95% CI: 1.5%, 28.5%] in the AI-CBT group to 13.8% [95% CI: 4.5%, 32.6%] in the AI-PDT group.

At the 1-month follow-up, response rates differed significantly across the three groups, χ^2^(2, *N* = 74) = 6.9, *p* = .03. However, follow-up pairwise comparisons using Yates' continuity correction showed that while the AI-PDT group (60.0% [95% CI: 36.4%, 80.0%]) significantly outperformed the Waitlist group (25.8% [95% CI: 12.5%, 44.9%]; *p* = .03), the difference between AI-CBT (52.2% [95% CI: 31.1%, 72.6%]) and the Waitlist did not reach statistical significance (*p* = .09). No significant difference in response rates was observed between the two active treatment protocols (*p* = .80). Remission rates at follow-up were also higher for the active treatments (25.0% [95% CI: 9.6%, 49.4%] for AI-PDT and 26.1% [95% CI: 11.1%, 48.7%] for AI-CBT) compared to Waitlist (9.7% [95% CI: 11.1%, 48.7%]), though this specific difference did not reach statistical significance (*p* = .22).

### 3.5 Predictors of treatment outcome

A multiple linear regression analysis was conducted to identify baseline predictors of post-treatment social anxiety severity among participants in the active treatment conditions (*n* = 38). To preserve statistical power given the sample size, the model was restricted to four clinically relevant predictors: baseline social anxiety (SPIN) and age (to account for baseline severity and potential generational differences in technology use), as well as the last therapeutic alliance (SAI) rating (week 4) and the presence of a neurodevelopmental diagnosis (ADHD/ASD).

The overall model explained a significant proportion of variance in post-treatment scores (R^2^__adj_ = 0.56, *F*[4, 33] = 12.5, *p* < .001). As expected, higher baseline severity significantly predicted higher post-treatment symptom severity (*p* < .001; *d* = 1.98). Notably, the presence of a co-occurring neurodevelopmental diagnosis was a significant predictor of outcome (Estimate = −6.59, *p* = .037). This corresponds to a moderate-to-large effect size (*d* = 0.76), indicating that neurodivergent participants finished treatment with significantly higher symptom burdens than their neurotypical peers, even after adjusting for baseline severity. Therapeutic alliance measured at week 4 showed a trend toward predicting better outcomes with a moderate effect size (*d* = 0.60), but this did not reach statistical significance (*p* = .09). Finally, age was not a significant predictor (*p* = .348; *d* = 0.33).

A sensitivity power analysis indicated that with *n* = 38 and four predictors, the model was adequately powered at 80% (alpha = 0.05) to detect a minimum effect size of Cohen's *d* = 1.20.

### 3.6 Treatment satisfaction and negative effects

Participants in both active conditions reported high levels of treatment satisfaction. There was no statistically significant difference in CSQ-8 total scores between the AI-PDT (*M* = 25.95, *SD* = 5.23) and AI-CBT (*M* = 25.35, *SD* = 4.44) groups, *t*(41) = −0.42, *p* = .68.

Regarding negative effects, a majority of the 45 participants who responded to the NEQ-20 reported at least one negative effect during treatment (76%). The most frequently endorsed items were “*Unpleasant memories resurfaced*” (23/45, 51%) and “*I experienced more unpleasant feelings*” (15/45, 33%). When restricted to effects participants considered likely caused by the intervention, the overall prevalence remained substantial (69% [31/45]). The specific items most frequently attributed to the treatment were the triggering of unpleasant memories (47% [21/45]) and increased unpleasant feelings (29% [13/45]). However, both the AI-PDT and AI-CBT groups reported similar frequencies of perceived negative events, with no significant difference between conditions (*t*(43) = 0.44, *p* = .66).

### 3.7 Process measures and weekly symptom monitoring

**Fig. 3:**
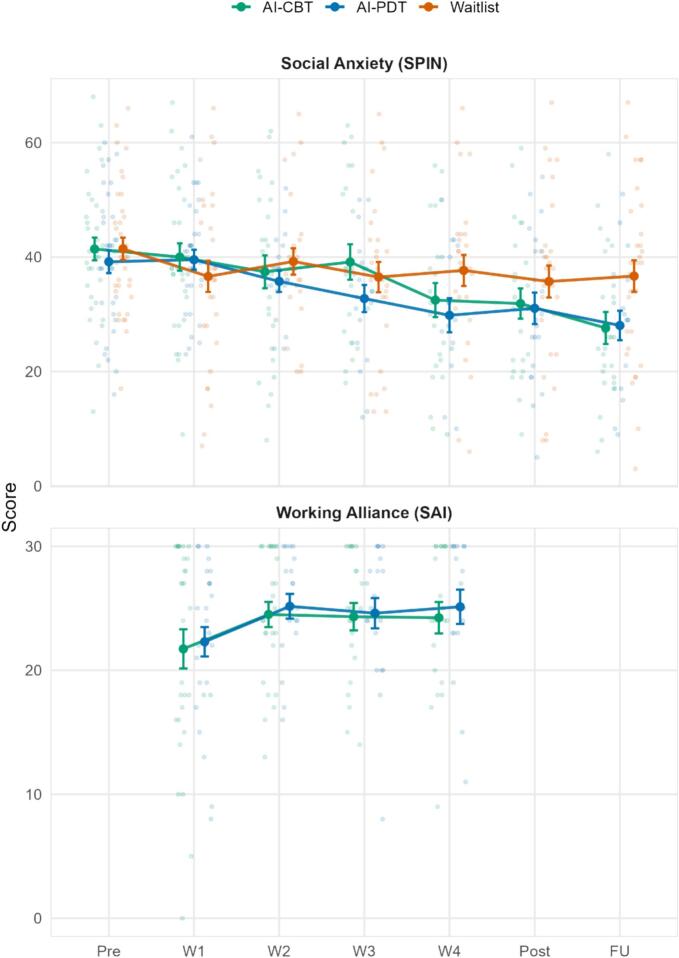
Weekly Trajectories of Social Anxiety and Working Alliance. *Note*. The top panel displays social anxiety symptom severity (SPIN) from pre-treatment through 1-month follow-up, including weekly monitoring (W1–W4) and the post-treatment assessment (Post). The bottom panel displays therapeutic working alliance (SAI) ratings reported weekly during the 4-week intervention. Faded points represent individual participant scores (time jittered for visibility); bold lines and error bars represent group means ±1 Standard Error (jittered for visibility).

To assess the trajectory of change and the quality of the therapeutic relationship, social anxiety symptoms (SPIN) and working alliance (SAI) were monitored on a weekly basis (see observed values in Fig. 3).

#### 3.7.1 Working alliance

Participants in both AI-guided conditions reported establishing a therapeutic alliance early in treatment (cf. Lindqvist et al., 2023). Mean SAI scores indicated a high working alliance starting from Week 1 (*M* = 22.3 for AI-PDT; *M* = 21.7 for AI-CBT) which was maintained and slightly increased through Week 4 (*M* = 25.1 for AI-PDT; *M* = 24.2 for AI-CBT). There were no observed differences in alliance trajectories between the psychodynamic and cognitive behavioral conditions, suggesting that the AI-delivered format facilitated comparable levels of engagement across modalities.

### 3.8 Participant perceptions of the AI

Weekly qualitative feedback provided insight into how participants experienced the AI assistant. Overall, participants across both active treatment groups highly valued the instant accessibility and non-judgmental nature of the AI, noting that it lowered the threshold for seeking support during acute anxiety. For instance, one participant in the AI-PDT group noted that “*It was easier to just be myself instead of adjusting what I say to match what I think the other person expects, which is what tends to happen when I talk to a human* (paraphrased)”. The AI-CBT group specifically praised the concrete, actionable steps provided, while the AI-PDT group highlighted the AI's ability to foster self-understanding and pattern recognition.

Regarding frustrations, participants noted that while the AI was highly accessible, instances where the AI forgot previously discussed information or repeated exercises were experienced as disruptive to the therapeutic alliance. Additionally, users expressed a desire for better organization of their exercises and, in some cases, for the AI to proactively set session boundaries to prevent overwhelm. For instance, one participant in the AI-PDT group noted that “*I'd like some built-in boundaries for daily and weekly usage. As it is now, the app just keeps responding no matter what, and that becomes overwhelming* (paraphrased)”.

A comprehensive descriptive summary of the qualitative weekly feedback, including group comparisons and numerous representative participant quotes, is available the *Supplementary Materials* (Table S2-S7).

## 4 Discussion

The aim of this randomized controlled trial was to evaluate the efficacy of two AI-guided smartphone interventions for social anxiety disorder: Psychodynamic Therapy (AI-PDT) and Cognitive Behavioral Therapy (AI-CBT). Contrary to our hypothesis, the intention-to-treat analysis did not reveal a statistically significant difference between the active interventions and the waitlist control at post-treatment assessment. This null finding appears driven by substantial improvement in the waitlist group (*d* = 0.54) combined with limited statistical power. While comparative efficacy was not established at post-treatment, there were some indications of a delayed treatment effect. At the 1-month follow up, AI-PDT demonstrated statistically significant superiority over the waitlist (*d* = 0.65), while AI-CBT showed a comparable moderate effect (*d* = 0.51), though it did not reach statistical significance. In terms of within-group improvement, both interventions yielded moderate symptom reductions from baseline to post-treatment (*d* = 0.79 for AI-PDT; *d* = 0.72 for AI-CBT), suggesting that while the distinction from waitlist was delayed, the interventions were clinically potent. However, given the strong control group response, these within-group reductions must be interpreted with caution, as a significant portion of the improvement may be attributable to the passage of time and measurement reactivity.

Regarding comparative efficacy, we found no significant differences between AI-PDT and AI-CBT in terms of symptom reduction, response rates, or therapeutic alliance. While our privacy protocols precluded the collection of objective usage logs, our qualitative findings indicated that AI-delivered interventions can effectively engage users on an experiential level, with high acceptability regardless of the underlying theoretical modality (see Supplementary Materials). The lack of significant differences in symptom reduction and alliance quality between AI-PDT and AI-CBT mirrors findings in adolescent internet-based treatments, where the alliance-outcome relationship was found to be equally important and stable across both PDT and CBT modalities (Lindqvist et al., 2023). This supports the notion that the working alliance is a ‘common factor’ that remains influential even when the delivery method shifts from human therapists to automated AI systems. Furthermore, our findings indicate that treatment response was independent of demographic factors such as age, which aligns with larger effectiveness trials of therapist-guided ICBT interventions (Niles et al., 2021). However, while the present analysis indicates potential utility and generalizability of AI-guided psychotherapeutic interventions, further research is needed to isolate the effects of the interventions from waitlist improvements before definitive claims can be made about clinical efficacy and broader implementation.

To provide clinical context, our follow-up between-group effects (*d* = 0.51–0.65) compare favorably to those typically reported for unguided self-help (Furmark et al., 2009; Mechler et al., 2024a). While these effects are understandably lower than the large pooled between-group effects (e.g., *g* = 0.95) established by meta-analyses of standard human-guided ICBT (Hall et al., 2025), achieving this magnitude of symptom reduction (within-group *d* = 0.72–0.79) in just four weeks demonstrates substantial clinical potency. This aligns with recent findings that brief digital formats can yield comparable clinical outcomes to standard-length interventions (Peynenburg et al., 2025), suggesting our automated 4-week AI-delivered treatment protocols provide a highly resource-efficient alternative. These effect sizes are also consistent with the outcomes reported in a recent meta-analysis of short-term psychodynamic psychotherapy for SAD (Norén et al., 2026), which found a large pooled effect of short-term psychodynamic psychotherapy versus waitlist (*g* = −0.97, k = 6) and no significant differences when compared to active treatments including CBT (*g* = 0.01, k = 9). The convergence between our AI-delivered PDT results and traditional therapist-delivered short-term psychodynamic psychotherapy outcomes is encouraging, though the treatment durations in the included short-term psychodynamic psychotherapy trials (8 to 31 sessions) were considerably longer than our 4-week format.

A notable finding was the substantial symptom improvement observed in the waitlist control group. While waitlist controls typically show minimal change in SAD trials (see e.g., Steinert et al., 2017), our control group demonstrated a medium effect size improvement (*d* = 0.54). This improvement is particularly striking given that waitlists are typically associated with symptom stability or even deterioration (17.4% per Rozental et al., 2017), rather than the significant gains observed here. Our findings thus suggest that the structured nature of weekly digital assessments may not only prevent such deterioration but actively facilitate early therapeutic gains; at least in the context of the 4-week intervention period of the present study (cf. waiting for treatment has negligible effects when the waiting duration is, on average, over 10-weeks which may explain the discrepancy between our post-treatment and follow-up assessment results; Steinert et al., 2017). The regular engagement required by weekly symptom monitoring and the expectancy of receiving a novel AI treatment may have functioned as a low-intensity intervention in itself; albeit this is speculative. That would align with the meaning-change hypothesis of measurement-induced improvement, which suggests that repeated assessment allows participants to discern the underlying construct more clearly, thereby shifting their interpretation and subsequent endorsement of symptom items (Knowles et al., 1996). Stated differently, one possible explanation is that the weekly monitoring protocol acted as a low intensity behavioral probe, inadvertently encouraging participants to monitor and confront their avoidance patterns; a core mechanism of change in SAD, even in the absence of formal therapeutic content (Adair, 1984; Enck and Zipfel, 2019; French and Sutton, 2010; Torous and Firth, 2016).

This high control response likely reduced the statistical power to detect between-group differences at post-assessment, particularly given that the effective sample size was reduced by attrition. However, it is crucial to note that as the transient effects of measurement reactivity likely faded, specific treatment effects emerged; at the 1-month follow-up, AI-PDT became significantly superior to the waitlist (*d* = 0.65), with a comparable effect size observed for AI-CBT (*d* = 0.51). Nevertheless, we do not currently have sufficient evidence to argue that these active interventions are definitively more effective than the passage of time plus regular measurement. Rather than proving robust clinical efficacy, the inclusion of a waitlist control was scientifically essential for highlighting this nuance. In the context of mobile health (mHealth) research, waitlist designs, while providing Class V comparison strength, are critical for evaluating intervention effects beyond the passage of time and regression to the mean (Goldberg et al., 2023). Without this control, the large within-group reductions in both AI-PDT and AI-CBT might have been erroneously attributed entirely to the active interventions, potentially overlooking the substantial contribution of measurement reactivity in digital trials. Finally, although all groups demonstrated symptom reductions, the underlying mechanisms likely differ. Waitlist improvements, driven by transient measurement reactivity, may be unstable and prone to relapse. In contrast, active interventions provide enduring psychological skills, theoretically fostering more stable, long-term change.

Secondary analyses identified a critical predictor of treatment response: the presence of a co-occurring neurodevelopmental diagnosis. Participants with a self-reported diagnosis of ADHD or ASD derived significantly less benefit from the AI interventions than neurotypical participants (a difference of approximately 6.6 points on the SPIN). This finding aligns with previous literature suggesting that the self-directed nature of digital interventions may pose challenges for individuals with executive function deficits (Grynszpan et al., 2014; Hollis et al., 2017). The lack of human co-regulation and the high demand for self-structure in AI-guided therapy may be particularly burdensome for this population, suggesting that future iterations of AI therapeutics require tailored scaffolding or hybrid human support to be effective for neurodivergent users.

Negative effects, particularly increased unpleasant feelings and resurfacing of memories, were common among participants who received treatment in our study. In automated formats, symptom activation can be therapeutically productive when appropriately paced and supported, but destabilizing without individualized titration or human co-regulation. These findings underscore the need for safety architecture in AI-guided interventions (Carlbring and Andersson, 2025), including proactive normalization of affective activation, structured check-ins when symptoms worsen, and clear escalation pathways to human support when distress exceeds predefined thresholds. That said, treatment satisfaction was generally high in both groups, exceeding treatment satisfaction ratings found in an outpatient setting (Pedersen et al., 2022).

Several limitations should be considered when interpreting these results. First, the study suffered from a high attrition rate, with approximately 30–35% of participants in the active conditions failing to complete the post-treatment assessment. While the Linear Mixed Model analysis accounted for missing data, the reduced sample size limited statistical power, particularly given the unexpectedly large improvement in the control group. Second, the intervention period was relatively brief (4 weeks). While sufficient to demonstrate feasibility and initial symptom reduction, a longer duration might be required to fully realize the benefits of psychodynamic working-through processes. Third, the absence of objective quantitative engagement metrics (e.g., login frequency or message counts) due to the privacy-by-design architecture meant that our assessment of engagement primarily relied on subjective participant feedback. Fourth, the interventions were designed as open-ended, on-demand tools, allowing users to interact at their own pace rather than progressing through a linear sequence of modules culminating in a traditional discharge phase. However, this meant that standard metrics of treatment completion are inapplicable to the present interventions. It should also be noted that the sample largely consists of highly educated, employed women. As such, more research is needed before concluding that the findings readily extend to other populations. Furthermore, reliance on self-report measures for the primary outcome introduces the potential for response bias, although the use of validated scales (SPIN, LSAS) allows for comparison with existing literature. Finally, no protocol or statistical analysis plan was publicly preregistered prior to data collection. While these limitations warrant caution when interpreting the results, our findings nevertheless provide preliminary insights into AI-guided psychological interventions.

## 5 Conclusions

This study demonstrates that AI-guided psychodynamic and cognitive behavioral interventions are feasible and capable of producing moderate-to-large reductions in social anxiety symptoms. While statistical superiority over a highly reactive waitlist was not established in the short term, the magnitude of within-group improvement over time suggests that AI-PDT and AI-CBT are clinically potent tools. The findings highlight the importance of accounting for measurement reactivity in digital trials and suggest the potential need to adapt AI-delivered treatments for neurodivergent populations. Future research should utilize active control conditions and larger samples to further disentangle the specific effects of AI guidance from general monitoring effects.

## Declaration of competing interest

Two of the authors (PC, GA) have published a self-help book that inspired the therapeutic approach and content delivered by the AI agent. All other authors declare no competing interests.
