# Supplementary material for: Anna vs. Judith: A randomized comparison of AI-delivered psychodynamic and cognitive behavioral therapies for social anxiety disorder
Source: Internet Interv. 2026 Jun 13;45:100960. doi: 10.1016/j.invent.2026.100960 (PMC13292244; doi:10.1016/j.invent.2026.100960)
Supplement: Supplementary material Table S1 [file mmc1.pdf]

Table S1. *Estimated Marginal Means (ITT) for Social Anxiety (SPIN) by Group and Timepoint.*

	Baseline		Post-Treatment		1-Month Follow-Up	
**Group**	**Mean (SE)**	**95% CI**	**Mean (SE)**	**95% CI**	**Mean (SE)**	**95% CI**
Waitlist	41.44 (2.20)	[37.08, 45.80]	34.81 (2.27)	[30.32, 39.30]	36.30 (2.24)	[31.86, 40.73]
AI-PDT	39.18 (2.20)	[34.82, 43.53]	29.53 (2.43)	[24.74, 34.33]	28.42 (2.48)	[23.53, 33.31]
AI-CBT	41.41 (2.20)	[37.05, 45.77]	32.65 (2.38)	[27.96, 37.34]	30.07 (2.40)	[25.34, 34.81]
