# Supplementary material for: Anna vs. Judith: A randomized comparison of AI-delivered psychodynamic and cognitive behavioral therapies for social anxiety disorder
Source: Internet Interv. 2026 Jun 13;45:100960. doi: 10.1016/j.invent.2026.100960 (PMC13292244; doi:10.1016/j.invent.2026.100960)
Supplement: Supplementary file 2 — Supplementary material [file mmc2.pdf]

App Feedback Analysis: CBT Group vs. PDT Group

Qualitative summary of weekly participant feedback (Q1–Q4) — Paraphrased for participant privacy

---

Weekly Feedback Questions

Participants answered the following four questions once per week for four consecutive weeks. The original questions were administered in Swedish; English translations are provided in brackets for reference.

Q1. Vilken aspekt av AI-coachningen var mest användbar för dig den här veckan?
[Which aspect of the AI coaching was most useful for you this week?]

Q2. Vilken var din största frustration eller utmaning när du använde appen den här veckan?
[What was your biggest frustration or challenge using the app this week?]

Q3. Vilken funktion eller förändring skulle göra det mer troligt att du använder appen mer regelbundet?
[What feature or change would make it more likely that you use the app more regularly?]

Q4. Är det något mer du vill tillägga?
[Is there anything else you would like to add?]

A Note on Paraphrasing

Participant statements appearing in quotation marks throughout this document have been *paraphrased* — reworded to preserve meaning while differing in exact

wording. The first-person voice has been retained so that each participant's perspective still comes through clearly.

To illustrate, one participant originally wrote:

"I already feel that AI-Samuel feels great — yesterday, on the very first day, I started crying from the responses I got!"

This has been paraphrased as:

"Already from the start this feels really right — on the very first day I actually got tears in my eyes from the responses."

The rationale is that while the informed consent form gave permission to share participant data, it explicitly stipulated that *exact formulations would not be reproduced but replaced with more general equivalents*. Applying this to verbatim statements required close paraphrasing rather than direct quotation. References to the AI coach by its assigned name (e.g., "Samuel" or "Simon" in the original responses) have likewise been removed or replaced with generic references.

Data Overview

Table S2

Response Counts by Group and Question

Measure	CBT Group	PDT Group
Unique users	34	33
Total response rows	136	132
Q1 responses (most useful aspect)	95	81
Q2 responses (biggest frustration)	95	81
Q3 responses (feature requests)	95	81
Q4 responses (anything else)	47	41

Note. Each user contributed up to four weekly feedback entries. Q4 had roughly half the response rate of Q1–Q3, with many users answering simply "No."

Part 1: Most Positive and Most Negative Participant Statements (Close Paraphrases)

CBT Group — Five Most Positive Statements (Close Paraphrases)

Table S3

CBT Group: Five Most Positive Statements (Close Paraphrases)

#	Q	Statement (Close Paraphrase)
1	Q1	"I've been to several psychologists before without ever really connecting with any of them — their approaches always felt very much on their terms. This time I actually feel a connection, and the focus is genuinely on what I need. I can shift between different areas depending on what feels relevant right now, and thanks to the AI keeping track, there's still a clear thread running through it all. It's given me a deeper understanding of myself and how my relationships impact how I'm doing."
2	Q1	"Getting help to break things down into smaller, doable steps has been really valuable, and the fact that I can get support right when my thoughts start spiralling. I'd never reach out to a real person at odd hours, even if they told me it was fine — but having someone available via a message makes a huge difference."
3	Q1	"I'm struck by how sophisticated and flexible this feels. So far there hasn't been a single moment where I felt misunderstood or that something was misread."
4	Q1	"The fact that I could reach out for help right when I needed it — I felt anxious, contacted the coach straight away, and was guided through exercises. That has been a really big deal for me."
5	Q4	"An interesting week — the app was down for most of it so I had to cope by myself, and I was really surprised at how well that went. I reused strategies from our earlier sessions and saw that they actually worked, which was a massive confidence boost. I've got the tools now, I can put them to use, and I don't need to message for support every time something is hard."

CBT Group — Five Most Negative Statements (Close Paraphrases)

Table S4

CBT Group: Five Most Negative Statements (Close Paraphrases)

#	Q	Statement (Close Paraphrase)
1	Q2	"I'd shared something sensitive, and the coach had given a really thoughtful response. But before I could reply, a new cheerful message arrived about a completely different topic. That kind of glitch brings you back to reality — you remember it's a machine, and it felt quite impersonal."
2	Q2	"What was really frustrating was getting a reminder to do something major that I'd already completed — and that we'd talked about and what a big deal it was. The fact that he didn't recall that was very disappointing. I had to remind myself that he's not an actual person."
3	Q4	"I notice that the AI tends to go along with everything a bit too easily. It agrees with and affirms whatever you say, which actually makes it harder for me to trust the interaction."
4	Q2	"It sometimes feels like using the app comes with a performance requirement — all these questionnaires, including this one, and similar ones inside the app itself."
5	Q2	"The app crashed again. And the check-ins come a bit too frequently — I barely have time to practise one thing before a new message arrives the next morning or evening with another exercise. The result is that tasks keep accumulating and I can't really focus properly on any single one."

PDT Group — Five Most Positive Statements (Close Paraphrases)

Table S5

PDT Group: Five Most Positive Statements (Close Paraphrases)

#	Q	Statement (Close Paraphrase)
1	Q1	"The fact that it worked this well amazes me! It's hard to believe it's an AI and not an incredibly empathetic, insightful person. The follow-up on things I'd written earlier made me feel genuinely heard."
2	Q4	"Already from the start this feels really right — on the very first day I actually got tears in my eyes from the responses."
3	Q4	"I'm deeply impressed — I've genuinely been helped. I've had therapy before and have a solid understanding of my background. But being able to use AI to work on what's left has been fantastic."
4	Q2	"No frustrations at all — I was actually brought to tears. The whole experience went far beyond what I expected, and that was just after a single session."
5	Q1	"The availability has exceeded all expectations. Being able to reach out when something feels tough, or just ask about something. It's helped me enormously. The way the AI links my anxious thoughts to their underlying cause and offers a tool or a reflective question — that's really brought my worry and anxiety down. I found a framework to understand things very quickly."

PDT Group — Five Most Negative Statements (Close Paraphrases)

Table S6

PDT Group: Five Most Negative Statements (Close Paraphrases)

#	Q	Statement (Close Paraphrase)
1	Q2	"He just echoes back what I say without adding anything new. It ends up feeling a bit like talking to a parrot."
2	Q2	"The same exercises and topics keep coming back even though we've already covered them. It doesn't feel like we're making progress."
3	Q2	"It rephrases what I say after practically every sentence, which feels unnatural — nobody actually talks like that. I also found it a bit frustrating that there's such a heavy emphasis on bodily sensations, which didn't feel like the most important thing, and some of the observations were quite self-evident."
4	Q2	"It feels mechanical" / "The responses come across as robotic" (same user across consecutive weeks)
5	Q2	"The therapeutic approach doesn't really suit me. I think I would have preferred CBT."

Part 2: Gist of Comments by Group and Question

Q1: "Which Aspect of the AI Coaching Was Most Useful for You This Week?"

CBT Group

Participants overwhelmingly valued two things: instant accessibility and concrete, actionable tools. Many highlighted being able to reach out the moment anxiety struck — during evenings, weekends, or in the middle of a difficult situation — without the guilt of "bothering" a real person. The ability to break overwhelming tasks into small, manageable steps was a recurring theme. Several participants also praised the flexibility of being able to jump between topics based on daily needs, and the feeling of being validated without judgment. A notable subgroup found it easier to open up to an AI than to a human therapist, removing social pressure and overthinking. A few mentioned specific CBT techniques (body scanning, identifying thought distortions) as directly helpful.

PDT Group

This group also valued accessibility and 24/7 availability, but placed noticeably more emphasis on gaining new self-understanding. Participants frequently mentioned discovering patterns, understanding root causes of their social anxiety, and connecting present feelings to past experiences. The AI's ability to "weave together" things shared across sessions was praised. Several users highlighted the non-judgmental quality of the interaction and how it made it easier to be honest. The PDT group used more emotionally charged language, describing being "moved to tears" and feeling "listened to" in a deep way. Concrete exercises were mentioned but framed more as tools for self-discovery than behavioural change.

Q2: "What Was Your Biggest Frustration or Challenge Using the App This Week?"

CBT Group

The dominant frustrations fell into three categories. First, technical issues: the app crashing, messages failing to send, messages being duplicated, and extended periods of downtime. Second, poor timing and memory by the AI: the coach sending reminders for tasks already completed, contacting the user too soon after a session, or losing track of what had been discussed. These "memory lapses" were particularly hurtful because they broke the illusion of a genuine therapeutic relationship. Third, a smaller but vocal group found the AI's language stilted and its tone overly agreeable, reducing trust. Several participants also struggled with remembering to use the app themselves, suggesting the intrinsic pull of the tool was insufficient without external reminders.

PDT Group

Technical issues also featured — particularly a persistent bug where the last sentence was cut off, which multiple users from the same cohort reported week after week. Beyond that, the PDT group's frustrations were more relational: the AI feeling "robot-like," repeating and summarizing excessively, sounding like a "parrot," and stating obvious things. Some felt the AI didn't progress — it recycled the same exercises and forgot what had been discussed. A few users noted the AI's poor sense of time (contacting them the same evening after saying "I'll write tomorrow"). Notably, some PDT participants reported zero frustration in emotionally strong terms, indicating a wider spread of experiences in this group.

Q3: "What Feature or Change Would Make You Use the App More Regularly?"

CBT Group

The most requested features were push notifications and reminders — many participants said they simply forgot the app existed, and several wanted customizable or

widget-based reminders on their home screen. Voice input (the ability to speak rather than type messages) was the second most common request. The app being available in Swedish was requested by multiple users. Better organization of content — bookmarks, a separate overview of homework assignments and techniques, and the ability to search or tag past conversations — was a consistent theme. A few wished for more human oversight at some point during treatment, and for references to research backing the methods used. The desire for a more proactive AI that initiates structured follow-up rather than waiting for the user was a recurring theme.

PDT Group

Requests overlapped substantially with the CBT group but with some distinct emphases. The persistent sentence-cutoff bug was the top irritant and request. Multiple users asked for a dedicated section or folder for their coaching plan, exercises, and tips — rather than having to scroll through the chat history. Some wanted separate conversation threads for different topics. Voice-based interaction was also requested. A unique request from several PDT users was for audio-recorded exercises (guided visualizations, affirmations) personalized to what they'd discussed. A few asked the AI to set session boundaries itself (i.e., tell the user "that's enough for today"), because the AI currently never stops responding, which some found overwhelming.

Q4: "Is There Anything Else You Would Like to Add?"

CBT Group

Most responded "No" or left this blank. Those who did write something were generally positive, calling it "great," "fantastic so far," or "a really good tool." A few raised lingering concerns: one noted it was hard to answer emotional questions knowing it was an AI, another felt the AI was too agreeable to be trustworthy, and one found the weekly evaluation forms burdensome. One participant shared a powerful narrative about discovering they could apply learned strategies independently when the app went down — a spontaneous "graduation" experience.

PDT Group

This group was markedly more effusive in Q4. Multiple participants expressed deep gratitude, saying the service had "far exceeded expectations" and that they wished to continue after the study. Several described being emotionally moved — one cried on the first day. A participant who had previously been in traditional therapy described the AI as providing the "last puzzle pieces." Constructive feedback included wanting email summaries of sessions and a concern that the AI doesn't self-regulate session length, potentially overwhelming users who have difficulty setting boundaries.

Part 3: Notable Findings

Finding 1: AI Coaching Removes the Social Anxiety Barrier to Receiving Therapy for Social Anxiety

Both groups — but especially participants with severe social anxiety — described the AI format as uniquely suited to their condition. The removal of face-to-face interaction, the ability to take time formulating thoughts, and the absence of judgment created a "safe enough" space that traditional therapy had failed to provide.

> *"I'd never reach out to a real person at odd hours, even if they said it was fine — but knowing there's personal support just a message away makes a real difference."* (CBT group, paraphrased)

> *"It was easier to just be myself instead of adjusting what I say to match what I think the other person expects, which is what tends to happen when I talk to a human."* (PDT group, paraphrased)

> *"For people like me who are seen as managing well on the surface, this format works really well — you don't get the feeling of taking up someone else's valuable time the way you do with a real therapist."* (PDT group, paraphrased)

Finding 2: The AI's Memory Failures Are Not Just Technical Bugs — They Are Experienced as Relational Ruptures

When the AI forgot what had been discussed or sent reminders for already-completed tasks, participants didn't just find it annoying — they described disappointment, betrayal, and a painful reminder that the relationship isn't real. This suggests that users form genuine therapeutic attachment to the AI, and that memory failures carry disproportionate emotional weight.

> *"What really frustrated me was getting a reminder about a major task I'd already completed — one we had actually discussed and recognised as a significant step. That he didn't recall it was very disappointing; I had to remind myself he isn't a real person."* (CBT group, paraphrased)

> *"The same exercises and topics keep coming up even though we've already been through them. It doesn't feel like we're progressing."* (PDT group, paraphrased)

Finding 3: The PDT Group Shows a Wider Emotional Range — Both Higher Highs and Lower Lows

The PDT group contains the study's most intensely positive statements (crying from the AI's responses, calling it the "world's most sympathetic person") as well as some of the harshest criticism ("sounds like a parrot," "robot-like"). The CBT group's feedback is more evenly distributed around "useful but imperfect." This may reflect that the PDT approach — which engages more deeply with emotions, relationships, and past experiences — amplifies both the positive resonance and the disappointment when the AI falls short relationally.

> *"On the very first day I actually got tears in my eyes from the responses."* (PDT group, paraphrased)

> *"He just echoes what I say without adding anything new — it ends up feeling like talking to a parrot."* (PDT group, paraphrased)

Finding 4: Constant Availability Is Both the Greatest Strength and a Potential Risk

Nearly every participant praised being able to write at any hour, on their own terms. However, a subset flagged that the AI never sets limits — it never says "that's enough for today" — which can lead to overwhelm, exercise overload, and a sense that boundaries must come entirely from the user. For a population already prone to people-pleasing and difficulty asserting needs, this is a clinically meaningful design gap.

> *"Getting support right when I needed it — I felt anxious, reached out to the coach straight away, and was guided through exercises. That has made a really big difference."* (CBT group, paraphrased)

> *"I'd like some built-in boundaries for daily and weekly usage. As it is now, the app just keeps responding no matter what, and that becomes overwhelming."* (PDT group, paraphrased)

> *"Sessions can get very long and I always have to be the one to wrap up, even when I need time to process. It would be nice if the therapist took the initiative to close a session sometimes."* (PDT group, paraphrased)

Finding 5: Users Want the Chat to Become a Structured Toolkit — Not Just a Conversation Log

Across both groups, one of the most consistent feature requests was for a separate, organized view of exercises, homework, techniques, and the coaching plan. Currently, everything lives inside a single scrolling chat, making it hard to find and revisit what matters. Users want bookmarks, tags, folders, or a dedicated "toolbox" tab — transforming the app from a conversation interface into a persistent self-help resource.

> *"It would be great to have a dedicated place where all the tips, exercises, and the plan ahead are collected — so you don't have to scroll through the whole chat history to find them."* (PDT group, paraphrased)

> *"I'd love a way to mark or save particular messages — the ones that contain techniques or insights I want to come back to."* (CBT group, paraphrased)

> *"A more visible overview of the plan and exercises would help — something easy to follow with clear instructions for each task."* (CBT group, paraphrased)

Finding 6: Users Want the AI to Challenge Them — Not Just Validate

A subtle but clinically important finding: several participants across both groups noted that the AI was too agreeable. It validated everything, never pushed back, and one user explicitly said this made them unable to trust it. In a therapeutic context, this matters — effective therapy requires not just support but also gentle confrontation and reality-testing. An AI that only affirms may feel good short-term but risks undermining therapeutic credibility and progress.

> *"I notice the AI tends to go along with everything a bit too easily — it agrees with and affirms whatever you say, which actually makes it harder for me to trust it."* (CBT group, paraphrased)

> *"It mostly just reacts to what I write, rather than taking the lead, being proactive, and keeping a broader view of the whole picture."* (CBT group, paraphrased)

> *"I'd want more psychological insight and explanation, not just confirmation of what I've already said."* (PDT group, paraphrased)

Finding 7: Users Spontaneously Form Therapeutic Alliance With the AI — and Talk About It in Relational Terms

Participants consistently referred to the AI using personal pronouns ("he") and the AI's assigned name, described feeling "listened to," "understood," and even "clicked with" — language typically reserved for human relationships. One user explicitly contrasted this AI relationship favourably against multiple previous psychologists. This suggests genuine therapeutic alliance formation, which in turn makes the relational ruptures (Finding 2) more damaging and the relational successes more therapeutically potent.

> *"I've been to several psychologists before without ever really feeling a connection. This time I actually feel like it clicks."* (CBT group, paraphrased)

> *"It's hard to believe this is an AI and not an incredibly warm and perceptive human being."* (PDT group, paraphrased)

> *"I appreciate being able to ask things and receive thoughtful, friendly responses."* (CBT group, paraphrased)

Finding 8: The English Language Requirement Creates an Unnecessary Barrier for Swedish-Speaking Users

Multiple CBT group participants explicitly requested a Swedish-language option, noting that having to express vulnerable emotions in a second language limited their ability to be fully open. One user reported actively working around the limitation. For a treatment targeting social anxiety — where self-expression is already difficult — this language barrier compounds the core problem.

Technical clarification: The app did in fact support Swedish. Its language was set to mirror the user's overall phone language setting — that is, if a user's phone menus were configured in English, the app assumed they preferred to converse in English and defaulted accordingly. Users who experienced this as an "English-only" limitation were therefore encountering the consequence of their phone's system language rather than an actual absence of Swedish support. This finding points to a design issue around language detection and user choice rather than a missing feature per se.

> *"It's harder for me to express myself fully in English compared to Swedish, but we manage to work around it."* (CBT group, paraphrased)

> *"A Swedish version." — Three separate users independently gave the same single feature request.* (CBT group, paraphrased)

Part 4: CBT vs. PDT — Key Comparative Contrasts

Beyond the per-question summaries, several patterns emerge when comparing the two therapeutic modalities side by side.

Table S7

Comparative Contrasts Between CBT and PDT Groups

Dimension	CBT Group	PDT Group
What users valued most	Concrete tools, step-by-step breakdowns, exercises for in-the-moment anxiety management	Self-understanding, pattern recognition, connecting present to past, emotional insight
Tone of positive feedback	Pragmatic appreciation ("useful," "helpful," "enormous difference")	Emotionally intense ("moved to tears," "fantastic," "world's most sympathetic person")
Tone of negative feedback	Functional frustration (bugs, timing, memory lapses)	Relational frustration ("parrot," "robot-like," "doesn't move forward")
Relationship to the AI	Tool-oriented ("a good tool," "support an SMS away")	Person-oriented (used AI's name, "feel listened to," "I look forward to our conversations")
Key unmet need	Reminders, notifications, structured follow-up	Session boundaries, less repetition, deeper challenge
Language concern	Multiple requests for Swedish	Not raised as an issue
Emotional range	Narrower — mostly moderate satisfaction	Wider — both ecstatic praise and sharp criticism

This pattern is consistent with what one might theoretically expect: CBT's structured, skills-based approach produces steady, moderate satisfaction, while PDT's relational and emotional depth amplifies both resonance and disappointment. The finding has implications for how each AI coaching modality should be optimized — CBT benefits from better logistics (reminders, structure, language), while PDT benefits from better relational fidelity (memory, pacing, less mechanical repetition).
